# Do Adolescents Adopt the Prosocial Behaviors of the Classmates They Like? A Social Network Analysis on Prosocial Contagion

**DOI:** 10.1007/s10964-024-02037-z

**Published:** 2024-07-04

**Authors:** Daniela V. Chávez, Diego Palacios, Lydia Laninga-Wijnen, Christina Salmivalli, Claire F. Garandeau, Christian Berger, Bernadette Paula Luengo Kanacri

**Affiliations:** 1 School of Psychology, Pontificia Universidad Católica de Chile, Santiago, Chile; 2 INVEST Research Flagship/Psychology, University of Turku, Turku, Finland; 3 Society and Health Research Center (CISS), Universidad Mayor, Santiago, Chile; 4 Millennium Nucleus for the Evaluation and Analysis of Drug Policies (nDP), Santiago, Chile; 5 Millennium Nucleus on Sociomedicine (SocioMed), Santiago, Chile

**Keywords:** Prosocial contagion, Peer influence, Peer status, Likeability, Prosocial behavior, Social network analysis, Susceptibility

## Abstract

While the influence of high-status peers on maladaptive behaviors is well-documented, socialization processes of prosocial behavior through high-status peers remain understudied. This study examined whether adolescents’ prosocial behavior was influenced by the prosocial behavior of the peers they liked and whether this effect was stronger when the peers they liked were also well-liked by their classmates. Three waves of data, six months apart, were collected among Chilean early adolescents who completed peer nominations and ratings at Time 1 (*n* = 294, Mage = 13.29, *SD* = 0.62; 55.1% male), Time 2 (*n* = 282), and Time 3 (*n* = 275). Longitudinal social network analyses showed that adolescents adopted the prosocial behavior of the classmates they liked - especially if these classmates were well-liked by peers in general. In addition, adolescents low in likeability were more susceptible to this influence than adolescents high in likeability. The influence resulted both in increases and – especially – decreases in prosocial behavior, depending on the level of prosociality of the liked peer. Findings suggest that likeability represents an important aspect of peer status that may be crucial for understanding the significance of peer influence with respect to prosocial behaviors during adolescence. Pre-Registration: https://osf.io/u4pxm.

## Introduction

In adolescence, engaging in prosocial behavior (i.e., voluntary actions undertaken to benefit others; Eisenberg et al., 2015) has been linked to increased social acceptance (Layous et al., 2012), higher friendship quality (Markievicz et al., 2001), a lower risk for internalizing and externalizing problems (e.g., Memmott-Elison et al., 2020) and better school performances (e.g., Caprara et al., 2000). It is therefore essential to identify social conditions that promote prosocial behaviors during adolescence, a period of heightened susceptibility to peer influence (Veenstra & Laninga-Wijnen, 2022). Peer influence ‒ adopting the behavior of other peers ‒ plays an important role in the development of aggression (e.g., Laninga‐Wijnen et al., 2017). Little is known, however, about the role of peers in prosocial behaviors. The few studies on this topic only considered the role of (best) friends (e.g., Crone et al., 2022), although adolescents may be influenced by peers other than their friends (Gommans et al., 2017), such as peers they like and would like to hang out with^1^. An important reason why adolescents may try to adopt the behaviors of the peers they would like to hang out with – henceforth referred to as “peers they like” for readability - is that it increases their chance to actually hang out or become friends with this peer (Bowker, 2004). Using a stochastic actor-oriented model (SAOM; Snijders et al., 2010) with three waves of data, the current study first aims to examine whether adolescents are influenced by the peers they like in their prosocial behavior. As not all peers are equally influential or equally susceptible to influence (Delay et al., 2022), it was also investigated whether these effects were more likely (1) when the peers whom adolescents liked were also well-liked by classmates in general, and (2) when the adolescents themselves were not well-liked by classmates in general.

### Peer Influence and Changes in Prosocial Behavior

Most peer influence studies on prosocial behavior have been based on friendship networks (i.e., friend ties), testing whether adolescents became similar in prosocial behaviors (or changed their behavior in a similar direction) to the ones they nominated as friends. However, the findings have been inconsistent: three social network studies on children and adolescents from three different cultures (i.e., Chilean, American, and Korean) found *no* evidence for friendship influence on prosocial behavior in early adolescents (Dijkstra & Berger, 2018; Molano et al., 2013; Shin, 2017), whereas three other studies detected significant influence effects but in two different directions. In two out of these three studies, they indicated that adolescents were more likely to decrease rather than increase prosocial behavior as a result of friendship influence (Laninga‐Wijnen et al., 2019; Logis et al., 2013). This influence of friendship may occur via various mechanisms. Adolescents may become more similar to their friends by spending much time together, observing their behavior, and mimicking each other consciously or unconsciously (perception-behavior paradigm; Chartrand & Bargh, 1999). It is also possible that friends mutually discuss how they should approach social situations and learn through mutual encouragement how they can socially interact with each other as well as with other peers (Laninga-Wijnen & Veenstra, 2021). Since friendship affiliation plays an important role during adolescence by providing a sense of belonging and emotional support (Hartup & Stevens, 1997), becoming more similar to one’s friends may be a way to maintain harmony in the friendship and preserve the friendship itself. Alternatively, they may adopt prosocial behaviors because they see their friends being rewarded (e.g., their friends receiving positive feedback or attention from peers) for their prosocial behavior.

Even though previous social network studies are valuable, they have overlooked the fact that adolescents are surrounded by peers other than their close friends (Gommans et al., 2017), who may also influence their prosocial behaviors. Some experimental work has examined this question by considering the role of anonymous peers. For instance, one study with Korean college students found that “anonymous confederates” in the immediate social context influenced students’ participation in a campaign by money donation. This suggests that even the prosocial behavior of *anonymous* peers - with whom adolescents do not have any relationship - exerted influence on the participants’ prosocial behavior (Park & Shin, 2017).

Adolescents might also be influenced by peers they *like*. Peer influence processes in liking networks may operate through mechanisms similar to friendships (e.g., imitation) but also through distinct ones, such as aspiration, where individuals conform themselves to the higher values of peers’ behaviors (Snijders & Lomi, 2019). For instance, adolescents may like peers who are not (yet) their friends but with whom they desire to affiliate or become friends. Indeed, peers who are liked by classmates appear to be highly sought after for friendships (Thomas & Bowker, 2013). This preference might be because liked adolescents tend to be prosocial and trustworthy (Parkhurst & Hopmeyer, 1998). Thus, conforming to these peers’ behaviors may be a way to develop a stronger connection with them, paving the way for friendship formation. As friendships tend to be relatively stable and formed with only a limited number of peers, conforming to the peers they like might extend adolescents’ opportunities for social connections and recreational activities. In addition, adolescents are particularly sensitive to peer reward regarding positive interactions (Cho & Hackel, 2022), and any sign of acceptance, especially from the peers they like, is probably important for their self-esteem and sense of belonging. Even without such reinforcement, aligning with the values and behaviors of peers they like and admire from a distance might help adolescents develop a favorable self-concept (Brechwald & Prinstein, 2011).

### Who Is More Influential? The Role of High-Status Peers

An important determinant of behavioral influence during adolescence is high status (Field et al., 2023). Research has highlighted two distinct aspects of social standing in the peer group: popularity and likeability. Popularity is a reputation-based type of peer status associated with more visibility within a peer group (Cillessen & Rose, 2005). Likeability, instead, reflects personal affection and is assessed as the degree to which an adolescent is well-liked and accepted by peers (Laursen et al., 2023). Popularity and likeability are not mutually exclusive, but they represent different types of status with different implications for behavior (van den Berg et al., 2020).

Adolescents’ tendency to conform to popular peers is well-established, but little is known about the influential power of highly liked peers. Well-liked peers may be attractive role models because they are well-embedded in the peer group and usually display positive behaviors that make them attractive peers with whom to interact and be affiliated (Thomas & Bowker, 2013). Additionally, well-liked peers may be considered role models particularly for adaptive behaviors because they distinguish themselves from other peers by being well-adjusted and displaying socially desirable behaviors that improve their well-being (de Bruine et al., 2019). Indeed, well-liked peers have been found to be stronger norm-setters for academic behavior than popular peers (Kwon & Lease, 2014). Moreover, as likeability is positively linked to altruistic prosociality and communal goals (Findley-Van Nostrand & Ojanen, 2018), well-liked students may be powerful role models for prosocial behavior, since the prosocial behaviors they display generally arise from a desire to benefit others without expecting personal gain (Costello & Zozula, 2018), and therefore may be seen as more *genuine* (Adler & Adler, 1995). Yet another reason for conforming to well-liked peers is the wish to increase one’s own likeability in the peer group, an argument often made for conforming to popular peers (e.g., basking in reflected glory; Dijkstra et al., 2010).

Despite the consistent positive association between likeability and prosocial behaviors found in the literature, little is known regarding the influential power of well-liked peers on others’ prosocial behavior. To date, only one experimental study with early adolescents has revealed that the intent to behave prosocially (e.g., volunteering) was stronger when such behavior was previously displayed by highly popular peers or by well-liked peers than when it was displayed by low-status peers (Choukas-Bradley et al., 2015): watching high-status e-confederates endorsing prosocial responses in hypothetical scenarios encouraged participants to increase their prosocial endorsement more as compared to watching low-status e-confederates endorsing prosocial behavior. Even though experimental designs provide accurate and reliable results by randomizing participants, their external validity is limited. Furthermore, when examining peer influence, it is important to prevent the misattribution of selection effects (i.e., the idea that similarly prosocial students are more likely to like each other) as social influence. This can be done by controlling for selection effects, which requires using a social network approach (Steglich et al., 2010).

### Susceptibility to Prosocial Peer Influence

Susceptibility to peer influence can be defined as the likelihood that peer conformity will occur (Laursen & Faur, 2022). In some cases, peer conformity occurs due to the characteristics of the influencer, but in other occasions, it is a product of the characteristics of the influencer and of the target of influence (Delay et al., 2022). Adolescence is an important period for peer influence because youth brains are still malleable and oriented toward the social environment, which facilitates behavioral modifications through peer influence (Telzer et al., 2018). However, not everyone is equally susceptible to peer influence (Laursen & Veenstra, 2023). For instance, adolescents with few alternative opportunities for friendships are more susceptible to the influence of friends (Faur et al., 2022), and younger adolescents are more susceptible to peer influence regarding prosocial behavior (Foulkes et al., 2018; Ahmed et al., 2020).

Because being liked by peers fulfills belonging needs (Baumeister & Leary, 1995), it is plausible that youth with lower levels of likeability are especially susceptible to the influence of highly liked peers. Less-liked adolescents have fewer friends (Stotsky & Bowker, 2018), and their susceptibility may be driven both by the desire to become more accepted by well-liked peers (the source of influence) and by the hope of being rewarded with more connections. Prosocial behavior is crucial for developing harmonious social relationships and may be especially important in adolescence when there is a heightened need for social belonging (Crone & Achterberg, 2022). Additionally, less-liked peers tend to be less prosocial (Eisenberg et al., 2015) and thus may have more ‘room’ to improve.

## The Current Study

Youth tend to adapt their behavior in response to the behaviors of their peers, particularly when these peers have a high status. However, peer influence has been mostly studied for negative behaviors (e.g., aggression), and when it comes to the contagion of positive behaviors such as prosociality, most research has focused on friendship networks. Little is known, however, about how peers can influence prosocial behavior in an adolescent’s likeability network (beyond friendship ties) and whether certain peers (i.e., well-liked students) may be particularly influential. This study examines whether adolescents increase (or decrease) in prosocial behavior if the peers they like are high (or low) in prosocial behavior. First, adolescents are expected to become more aligned in prosocial behaviors with the peers they like (Hypothesis 1), particularly if those peers are also well-liked by the peer group in general (*social reinforcement hypothesis*, Hypothesis 2). Second, adolescents low in likeability are expected to be more susceptible than well-liked peers to this influence (Hypothesis 3). These influence processes are expected to occur after controlling for selection effects. Regarding selection effects, prosocial adolescents are expected to attract more liking nominations (Hypothesis 4). This study is the first to test susceptibility to peer influence regarding prosocial behavior while focusing on liking nominations, using peer-rated prosocial behaviors, and applying a longitudinal social network approach.

## Method

### Participants and Procedure

This study uses longitudinal data collected as part of a larger research project testing the effects of an intervention designed to promote prosocial behavior and social cohesion among elementary school students in Santiago, Chile (for details, see Luengo Kanacri & Jiménez-Moya, 2017; Luengo Kanacri et al., 2020). Participating schools were selected according to socioeconomic heterogeneity criteria to incorporate students from different socioeconomic backgrounds and were randomly assigned to the intervention (four schools; *n* = 336) and control (four schools; *n* = 324) conditions. Considering that one of the main outcome variables was prosocial behavior, only data from the four control schools was analyzed. The data, including three waves of measurement, were collected in May 2017 (nT1 = 294), October 2017 (nT2 = 282), and May 2018 (nT3 = 275). Classroom size ranged from 36 to 45 students at T1 and T2, and from 33 to 44 at T3. Participation rate per classroom ranged from 95 to 100% at T1, from 93 to 100% at T2, and from 98 to 100% at T3. As the academic year starts in March in Chile, the first two waves of data were collected when students attended the 7th grade (Mage = 12.31, *SD* = 0.58; 56.3% males), and the third (and last) wave of data was collected when students attended 8th grade (Mage = 13.29, *SD* = 0.62; 55.1% male). Students’ ages ranged from 11 to 15 years at T1. Seven classrooms participated in the study. Among the students, 20.4% belonged to the middle class, 16.4% to the low-middle class, 7.4% to the low class, and 0.6% belonged to the middle-high class. For 55.2%, no information on SES was available. About 50.3% of students were Chilean, 13.9% were from Perú, 1.5% from Bolivia, 2.8% from Venezuela, 1.9% from Colombia, and 29.6% of this information was missing.

Participants received active parental informed consent and gave their own assent. The reasons for attrition were, in most cases, related to students’ absence on the day of data collection due to illness. The questionnaires were designed to take approximately 30 min to complete and were administered in each classroom by three to four members of the research team during school hours. The response choices of the questionnaires were explained to students during data collection. For the sociometric question, participants received a roster with the names of all students in their classroom and were instructed to nominate up to three who best fit the description. Both same- and cross-sex nominations were allowed. Self-nominations were discouraged during testing and discarded during data processing. All instruments and procedures were approved by the ethics committee at the Catholic University of Chile and by the Chilean National Funding of Science and Technology (FONDECYT).

## Measures

### Likeability Network

To assess the *Likeability Network*, at each observation, students were asked, “*With whom would you like to hang out at school during recess?*”. Using a classroom roster, adolescents were allowed to nominate up to three (same or other sex) peers. Adjacency matrices were created for each classroom based on these nominations, with 0 = no nomination and 1 = nomination from one peer (in the row) to another peer (in the column). Missing data was coded as −99 for random missingness (e.g., did not attend the day of data collection), and 10 when it referred to the impossible nomination (e.g., students who had left the school).

### Prosocial Behavior

Individual prosocial behavior was measured with peer ratings. At each wave, the participating students were asked to rate the frequency of four representative types of prosocial behavior (“He/she tries to comfort other classmates when they are sad”; “He/she shares with others things he/she likes”, “He/she tries to understand the point of view of others”; “He/she helps others who are in need or have problems”) displayed by each of their classmates on a five-point scale ranging from 1 (never) to 5 (almost always). A score of prosocial behavior was computed for each individual by averaging the ratings they received from all classmates, across the four items. The Cronbach’s alpha coefficients showed high reliability at each time point (T1 α = 0.96; T2 α = 0.95; and T3 α = 0.95). Since RSiena cannot properly handle continuous measures (Ripley et al., 2023), students’ average prosocial behaviors were recoded from 0 to 4 and categorized into quintiles based on the entire sample across three waves (category 0 = 2.69 or lower, category 1 = 2.70–3.06, category 2 = 3,07–3.43, category 3 = 3.44–3.81, category 4 = 3.82 or higher) to be incorporated into the models.

### Sex

Students reported their sex at the beginning of the questionnaire by answering the question “What gender do you identify with”? Sex was coded as 0 = Girl; 1 = Boy.

### Analytical Strategy

#### Longitudinal Bayesian Social Network Analysis

To examine the selection and influence of liked peers regarding prosocial behavior, a longitudinal social network analysis was conducted, and implemented in the *RSienaTest* package (version 1.2–12) in R (version 3.5.1). Social network analyses (or stochastic actor-oriented models; SAOMs) enable us to investigate whether similarity in prosocial behavior among peers in a liking network is due to selection (similarly prosocial peers liking each other) or influence (peers adopting the prosociality of the classmates they like), while controlling for structural network effects and the general development of prosocial behavior. In detail, SAOMs model the overall change in a network and behaviors as a result of repeated micro steps in which actors change one network tie (e.g., likeability tie) or one behavioral level (e.g., prosocial behavior) at a time. In each micro-step, a randomly chosen actor decides to create a new network tie, drop an existing one, or not make a change. In each micro-step, a randomly chosen actor might increase, decrease (by one unit), or maintain his/her behavior.

In order to achieve the necessary statistical power and to account for potential heterogeneity between classrooms, multilevel random coefficient models (Snijders et al., 2010) with Bayesian estimation methods (sienaBayes function; Ripley et al., 2023) were applied. Bayesian inference assigns prior probability distribution to parameters and is updated to posterior probability distributions by observing the data. Posterior means and standard deviations for the fixed parameters *η* and the random parameters *μ* are estimated. Moreover, *p*-values of the parameter estimates are generated, which indicate the posterior probability that the parameter is greater than zero. This reflects the percentile where zero is located at the posterior distribution. The chances of the parameter being smaller than 0 can be retrieved by [1–*p*]: *p*-values of ≥0.95 and ≤0.05 indicate a high posterior probability that the alternate hypothesis is true.

In the models, parameters corresponding to the hypotheses (related to prosocial behavior selection and influence effects) were assumed constant across classrooms to gain power, whereas structural network effects (such as reciprocity and covariates effects) were allowed to vary randomly between classrooms. Prior knowledge based on earlier studies using liking networks in adolescents was incorporated (Mikami & Mercer, 2017; Sentse et al., 2014; Van Ryzin et al., 2016). The comparison between analysis with priors and an analogous model without prior mean information for the first model can be seen as a supplementary analysis in Table 3 (see Appendix A).

In the SAOM analysis, up to 20% of missing data is allowed for network variables and covariates. In RSiena, missing data is handled by internal imputation procedures that minimize their impact on parameter estimation (for details, see Huisman and Steglich, 2008). All covariates were centered in the analyses. Multiple sequences produced independently for assessing convergence were estimated (Gelman et al., 2014). For that, the function monitor implemented in the rstan R-package was used. To improve convergence, an increase in iterations was needed, using the parameters *nmain* and *nwarm* to 2000 and 500, respectively. Finally, all the parameters of interest presented an Rˆ ≤ 1.1, indicating good convergence (Rˆ indicates the potential scale reduction of the posterior distribution if simulations were continued indefinitely). All final models converged well according to standard convergence assessments for random-coefficients multilevel SAOM (Ripley et al., 2023).

## Model Specification

### Structural Network Effects

The following effects were included to capture the basic tendencies of actors to form and maintain liking relationships. *Density* describes the tendency of actors to give liking nominations. *Reciprocity* is the tendency to reciprocate liking nominations (referring to forming mutual liking ties). *Transitivity* was measured by including the tendency of students liking classmates who are liked by the peers that I like (transitivity GWESPFF). Regarding degree-related effects, the *indegree-popularity* and *outdegree-popularity* effects were included to represent the tendency of actors who receive many liking nominations to receive even more liking nominations over time, and actors who send many liking nominations to receive more liking nominations over time, respectively. Table 4 in Appendix B summarizes the RSiena effects and parameters included in the models.

### Covariates

Sex and prosocial behavior were included as covariates by including the selection effects for each. Three selection dynamic effects (prosociality alter, prosociality ego, prosociality ego*alter) and three selection constant effects (*sex alter, sex ego, same-sex*) were included. The alter and ego effects capture the effects of covariates on received and given nominations, respectively. The same-sex effects capture students’ tendency to befriend (or stay friends with) same-sex peers, and the prosocial ego*alter effect examines whether students with similar prosocial behaviors are likely to become or remain friends.

### Influence Effects

To accurately estimate the influence of (well-)liked peers on prosocial behavior, the prosocial behavior linear and quadratic shape effects were added to control for them. A positive linear shape effect expresses a primary drive toward high values on prosocial behavior. For the quadratic shape effect, a positive parameter suggests a self-reinforcing tendency and a negative parameter indicates a regression to the mean. Furthermore, the prosocial behavior indegree effect was included to represent the tendency of highly liked peers to increase in prosocial behavior over time. The main effect of sex on prosocial behavior was also incorporated. Initially, the analyses also included a *prosocial outdegree* effect, but this one was left out because of convergence issues. These convergence issues most likely occurred because the liking nominations were limited to up to three classmates.

Based on the hypotheses, three different models were estimated. In the first model, the hypothesis that adolescents are more likely to adopt the prosocial behaviors of the peers they like (*average alter effect*) was tested. The second model tested the hypothesis that adolescents’ tendency to adopt the prosocial behaviors of the peers they like is particularly strong if these peers are well-liked by others. In this model, the *popAlt* parameter describes the average indegree of liking nominations for the peers that adolescents like, and the *avAltPop* parameter, which is the interaction effect of the *average alter effect* and *popAlt* effect, were included. The third model tested whether peer influence in prosocial behavior varies as a function of adolescent’s own likeability levels. Adolescents low in likeability were expected to be more susceptible to being influenced by prosocial behavior than those high in likeability. To test this model, a parameter estimating the association between adolescents’ own likeability (*indeg*), as well as an interaction term to assess whether peer influence in prosocial behavior was moderated by the adolescents’ own likeability (*indeg x average alter*) were added. Finally, the last hypothesis that prosocial adolescents would attract more liking nominations, was tested with selection effects included in all the models as control effects (*prosocial alter effect*).

## Results

### Descriptives

Table 1 describes the changes and stability in liking networks across the three waves. The average Jaccard Index was about 0.25, indicating sufficient stability for conducting a social network analysis (Veenstra et al., 2013); yet it should be noted that the Jaccard Index was below 0.20 for some classrooms. The average number of liking nominations was around 2.4 in the first and second waves and slightly decreased to 2.27 in the third wave.

**Table 1:** Description of Liking Networks and Prosocial Behavior per (and across) Time Point(s)

Class	N	Liking Jaccard		Liking av. degree			Liking ties changes t1–t2			Liking ties changes t2–t3			Prosociality			Prosociality changes t1–t2			Prosociality changes t2–t3		
		t1–t2	t2–t3	t1	t2	t3	0→1	1→0	1→1	0→1	1→0	1→1	t1	t2	t3	dw	up	con	dw	up	con
5A	43	0.33	0.23	2.58	2.65	2.34	50	50	49	48	68	34	3.12	3.05	2.97	7	3	29	10	3	22
6A	40	0.32	0.28	2.51	2.62	2.18	38	40	37	35	48	33	2.69	2.59	3.47	9	7	20	2	22	9
6B	39	0.26	0.26	2.98	2.99	2.40	56	56	40	47	57	36	3.21	3.29	3.08	4	7	26	10	3	20
7A	50	0.33	0.32	2.33	2.32	2.33	46	51	47	38	55	44	2.07	2.64	2.41	2	17	17	9	4	21
7B	47	0.21	0.16	2.66	2.40	2.50	63	75	38	60	80	26	2.24	2.09	2.68	11	7	25	1	16	20
7C	51	0.19	0.27	2.11	1.92	2.28	53	60	27	33	53	31	2.28	2.43	2.57	6	7	21	3	12	16
8A	51	0.16	0.22	1.77	1.72	1.84	38	52	17	34	46	22	2.07	2.97	3.22	2	25	11	3	13	16
Av/Sum	321	0.26	0.25	2.42	2.37	2.27	344	384	255	295	407	226	2.53	2.72	2.91	41	73	149	38	73	124

Jaccard index refers to tie stability between observations; dw: number of actors who decreased his/her prosociality in this period; up: number of actors who increased his/her prosociality in this period; con: number of actors who remained his/her prosociality constant in this period

### Selection Effects Based on Prosociality

Table 2 provides the findings for selection processes based on prosocial behavior. As expected, results in all the models show that highly prosocial adolescents received more liking nominations (prosociality alter effect, [*η* = 0.22, SD = 0.03, *p* > 0.99] for Model 1, [*η* = 0.22, SD = 0.04, *p* > 0.99] for Model 2, and [*η* = 0.22, SD = 0.03, *p* > 0.99] for Model 3). With regard to control effects, a strong and significant tendency toward reciprocity and transitivity in adolescents’ liking networks as well as a tendency toward same-sex liking nominations (positive same-sex effect) were found. Moreover, adolescents tended to like peers with similar prosocial behavior levels (positive prosocial similarity effect).

**Table 2:** Longitudinal Bayesian Social Network Analyses on a Liking Selection and Influence Associated to Prosocial Behavior Across All Classrooms (*N* = 7)

Parameters	Model 1					Model 2					Model 3				
	Est.	SD	*p*	95% CI		Est.	SD(ƞ)	*p*	95% CI		Est.	SD(ƞ)	*p*	95% CI	
*Network & Selection Effects*															
Outdegree (density) (ƞ)	−1.90	0.14	0.00	−2.19	−1.63	−1.87	0.13	0.00	−2.18	−1.59	−1.92	0.13	0.00	−2.19	−1.67
Reciprocity (ƞ)	1.04	0.11	>0.99	0.80	1.29	1.07	0.13	>0.99	0.83	1.29	1.05	0.12	>0.99	0.82	1.29
Transitivity (gwespf FF) (ƞ)	1.20	0.11	>0.99	0.99	1.41	1.20	0.13	>0.99	0.97	1.45	1.18	0.12	>0.99	0.95	1.41
Indegree-popularity (ƞ)	−0.02	0.06	0.35	−0.15	0.10	−0.03	0.06	0.30	−0.15	0.09	−0.03	0.06	0.33	−0.16	0.10
Outdegree-popularity (ƞ)	−0.07	0.08	0.19	−0.21	0.09	−0.08	0.08	0.15	−0.22	0.07	−0.06	0.08	0.22	−0.20	0.09
** Prosociality alter** (μ)	**0.22**	**0.03**	**>0.99**	**0.16**	**0.29**	**0.22**	**0.04**	**>0.99**	**0.15**	**0.30**	**0.22**	**0.03**	**>0.99**	**0.16**	**0.28**
Prosociality ego (μ)	−0.06	0.03	0.01	−0.12	−0.01	−0.08	0.03	0.00	−0.13	−0.02	−0.07	0.03	0.00	−0.13	−0.01
Prosociality similarity ego*alter (μ)	0.06	0.02	>0.99	0.02	0.11	0.07	0.03	>0.99	0.03	0.12	0.07	0.02	>0.99	0.02	0.12
Sex alter (ƞ)	0.13	0.09	0.92	−0.05	0.32	0.13	0.10	0.92	−0.06	0.32	0.13	0.09	0.92	−0.05	0.31
Sex ego (ƞ)	0.01	0.10	0.54	−0.18	0.21	0.01	0.09	0.53	−0.18	0.19	0.01	0.09	0.54	−0.17	0.19
Same sex (ƞ)	0.32	0.11	>0.99	0.11	0.53	0.32	0.11	>0.99	0.07	0.50	0.32	0.10	>0.99	0.12	0.52
*Influence effects: Prosocial behavior*															
Prosociality linear shape (ƞ)	−0.51	0.23	0.01	−0.94	−0.06	−0.48	0.17	0.00	−0.85	−0.17	−0.98	0.38	0.00	−1.99	−0.35
Prosociality quadratic shape (ƞ)	−0.27	0.09	0.00	−0.46	−0.09	−0.20	0.08	0.01	−0.36	−0.03	−0.31	0.10	0.00	−0.52	−0.11
Prosociality indegree (ƞ)	0.22	0.11	>0.98	0.01	0.42	0.20	0.08	>0.99	0.04	0.36	0.41	0.16	>0.99	0.12	0.79
Sex (ƞ)	−0.37	0.19	0.03	−0.74	0.01	−0.37	0.15	0.00	−0.70	−0.08	−0.41	0.22	0.03	−0.87	0.00
** Prosociality average alter** (μ)	**0.64**	**0.20**	**>0.99**	**0.25**	**1.05**	**0.31**	**0.14**	**>0.99**	**0.08**	**0.59**	**1.55**	**0.50**	**>0.99**	**0.69**	**2.81**
** Prosociality average alter x popularity alter** (μ)	^**-**^	**-**	**-**	**-**	**-**	**0.45**	**0.16**	**>0.99**	**0.18**	**0.71**	–	–	–	–	–
** Prosociality average alter x Liking indegree** (μ)	^**-**^	**-**	**-**	**-**	**-**	**-**	**-**	**-**	**-**	**-**	−**0.35**	**0.17**	**<0.02**	−**0.75**	−**0.05**

P-values represent the percentile of zero in the posterior distribution. *P*-values of ≥0.95 and ≤0.05 reflect a high posterior chance that the alternate hypothesis is true. The rows in bold represent the estimates regarding the main hypotheses

*Est.* estimated for posterior means, which is fixed (ƞ) for control variables and random (μ) for variables testing the hypotheses, *SD(ƞ)* posterior standard deviation, *CI* credibility interval

### Do Adolescents Adopt the Prosocial Behavior of the Classmates They Like?

The negative linear shape of prosocial behavior indicated an overall tendency to lower levels of prosociality over time, and the negative prosociality quadratic shape indicated that prosocial scores tended to regress to the mean. As indicated by the positively *average alter* effect for prosocial behavior (see Table 2, model 1), in line with Hypothesis 1, youth adopted the levels of prosocial behavior to the peers they liked [*η* = 0.64, SD = 0.20, *p* > 0.99]. An ego-alter influence table was constructed to evaluate whether this effect was driven by the tendency to increase one’s prosocial behaviors in response to liked peers’ high prosocial behavior, or to decrease one’s prosocial behavior in response to liked peers’ low prosocial behavior (see Table 5 in Appendix C). Table 5 indicates that adolescents increased and decreased in their prosocial behavior influenced by the peers they liked. Comparing the values between rows and columns indicates that students mostly moved toward the lower prosocial behavior of their liked peers. However, those who were already relatively high on prosociality (scoring on average 2, 3, or 4 in prosocial behavior), still increased their prosocial behavior as a function of the prosocial behavior of their liked peers. Next, in line with Hypothesis 2, the *avAltPop* parameter in Model 2 indicates that adolescents’ tendency to adopt prosocial behaviors of the peers they liked was especially strong if these peers were highly liked by others [*η* = 0.45, SD = 0.16, *p* > 0.99]. Finally, Model 3 indicates that less-liked adolescents were more susceptible than well-liked adolescents to be influenced in prosocial behavior by the classmates they liked [average alter x indegree: *η* = −0.36, SD = 0.17, *p* = 0.02].

### Sensitivity Analysis

The avAltPop parameter provides information on whether students change their prosocial behaviors in a similar direction as the prosocial behavior of well-liked peers, however, the direction of these changes – that is, towards higher or lower prosocial behavior – remains unknown. For this reason, it was examined whether these well-liked peers were more prosocial as compared to less-liked peers as an exploratory sensitivity analysis. This would provide some indication of the direction of influence processes.

Since the peer nomination question associated with peer status was used in this study as a likeability network (“*With whom would you like to hang out with”*), a continuous variable was created for peer likeability based on the proportion scores for each student. The variable was then categorized into three different levels (low, middle, high) to test whether highly liked adolescents (1 SD upper mean) were more prosocial than those scoring middle and low in likeability (1 SD lower mean) across all measurement waves. A series of one-way ANOVAs showed significant differences between the three groups at T1, F(2, 278) = 26,38, *p* < 0.001; T2, F(2, 278) = 30,98, *p* < 0.001, and T3 F(2, 270) = 20,97, *p* < 0.001. Bonferroni post-hoc comparisons showed that students scoring higher in likeability were significantly more prosocial than those with middle (Δ = 0.31, SE = 0.07, *p* < 0.001) and low levels of likeability at T1 (Δ = 0.71, SE = 0.09, *p* < 0.001). At T2, the high-likeability group was also more prosocial than the middle (Δ = 0.43, SE = 0.07, *p* < 0.001), and the low-likeability group (Δ = 0.73, SE = 0.09, *p* < 0.001); and the same results were found at T3 comparing the highest with the middle (Δ = 0.41, SE = 0.08, *p* < 0.001), and highest with the lowest group (Δ = 0.58, SE = 0.09, *p* < 0.001).

## Discussion

Research on peer relations has suggested that youth are more likely to conform to the behavior of high-status, as compared with low-status peers (Gommans et al., 2017), and this has mainly been studied for undesirable outcomes such as risk attitudes (Rambaran et al., 2013) and aggressive behaviors (Laninga‐Wijnen et al., 2017; Laninga‐Wijnen et al., 2019). Whereas the influence of peers on prosocial behavior has also received attention in the literature, the persistent focus on friendship ties in studies on peer influence processes implies that the potential role of other types of peer relationships has been overlooked. Using a longitudinal social network design, this study showed that peer influence on prosocial behavior took place in adolescents’ liking networks. Specifically, after controlling for selection effects, students aligned their own prosocial behavior to the prosocial behavior of the classmates they liked. The influence on prosocial behavior was particularly driven by highly liked peers (i.e., those who were also liked by the peer group in general).

Adolescents low in likeability were more susceptible than highly liked ones to the influence of the peers they liked. Higher susceptibility to peer influence for low-status individuals has only been documented for undesirable behaviors (i.e., alcohol misuse) in a social network study (DeLay et al., 2022). The present study extends upon previous research on susceptibility to peer influence by showing that conformity to desirable behaviors, such as prosociality, can also depend on the status of the target of influence.

### Prosocial Peer Influence: Conforming to the Peers One Likes

This study demonstrated that adolescents modified their prosocial behaviors in a similar direction as the classmates they liked, finding support for contagion processes in prosocial behavior based on their liking preferences. These influence processes could go in two directions, that is, an *upward influence* when adolescents become more prosocial over time, and also a *downward influence* when adolescents become less prosocial over time to resemble the peers they like. The findings showed that those who were already prosocial (middle or high) still increased their prosocial responses as a function of the prosocial behavior of their liked peers. However, consistent with a previous study (Laninga‐Wijnen et al., 2019), the tendency to become less prosocial based on the behavior of peers was even more likely to occur. This downward influence can probably, to a large extent, be explained by similar mechanisms as the upward influence, such as the desire to affiliate with, befriend, receive positive rewards from the liked peer, or feel good about being similar to them. However, taking into consideration the normative decline in prosociality during this developmental period (Carlo et al., 2007; Luengo Kanacri et al., 2013), perhaps refraining from prosocial behavior can be considered an active (and even valued) choice as much as engaging in it. It should also be noted that anyone can refrain from prosocial behavior, whereas being prosocial requires socio-cognitive skills, such as the capacity to understand others’ views and needs and to adjust one’s behavior to situational demands. Therefore, it may be challenging for adolescents with low levels of prosocial behavior to increase their prosociality (to the extent that peers would notice the change and report it) if they lack such skills. There may be a statistical reason as well: adolescents’ scores in prosocial behavior in this study were average to high. Therefore, the chance for changing their behavior in a downward direction was more likely to happen than the chance for changing their behavior even more upward.

Past research has predominantly focused on friendship networks when studying the socialization of prosocial behavior. Thus, these findings extend upon previous work by demonstrating that the tendency to modify one’s behavior to match peers’ behavior does not only occur within friendships but in liking networks too. Peers’ likeability reflects their acceptance in the peer group (Gommans et al., 2017), and even though likeability and friendship networks might overlap to some extent, research has shown that many adolescents desire to be friends with highly liked peers (Thomas & Bowker, 2013), suggesting that liked peers are youth with whom adolescents would like to develop a friendship in the future. However, adolescents’ friendships are intimate bonds that provide them with emotional support and fulfill their need for trust, intimacy, and attachment (Hartup & Stevens, 1997), which liking preferences might not fulfill. Therefore, these results provide important knowledge for understanding the socialization of prosocial behavior during adolescence based on liking ties, confirming that the influence of peers on prosocial behavior in a sample of early adolescents resulted from the heightened influential power of highly liked adolescents and the heightened susceptibility of less-liked peers.

### “The Prosocial Influencer”: A Positive Reputation Leading to Contagion Processes

This study highlights the role of well-liked peers in the socialization of prosocial behavior. That is, adolescents adopt the prosocial behavior of the peers they like, especially when these peers are also highly liked by their peer group. Theories of instrumental learning suggest that actions that produce positive and rewarding outcomes are more likely to be adopted compared to unrewarding and punishing actions (Cho & Hackel, 2022). This learning is characterized by models of social reinforcement; that is, when adolescents receive positive feedback and rewarding outcomes (e.g., being liked) after performing a prosocial action, this will guide intentions and behaviors. The sensitivity analysis confirmed that highly liked peers were rated as significantly more prosocial than those scoring average and low in likeability, suggesting that the prosocial influence of well-liked peers was in the upward direction. This increase is potentially driven by an *aspiration* dimension, in which individuals increase their behaviors to conform to the higher values of other peoples’ behavior (Snijders & Lomi, 2019). Thus, adolescents who sent more liking nominations to peers with higher levels of prosocial behaviors and, as a result, increased prosociality themselves might perceive anticipated rewards associated with more connections and friendship opportunities with these peers. Additionally, they might expect that behaving similarly to well-liked peers will help enhance their own status (basking in reflected glory, Dijkstra et al., 2010) or gain more acceptance from peers (Chávez et al., 2022). This is consistent with other research suggesting that anticipating social acceptance feedback increases activation in brain regions linked to both reward processing and social cognition (Powers et al., 2013). If rewarding outcomes shape affect and prosocial behaviors (Cho & Hackel, 2022), this positive cycle may encourage other adolescents to engage in the same behavior via social reinforcement, especially when the social reinforcement is from a valued peer (Bandura & Walters, 1977).

The motivations underlying these influence processes remain to be investigated. Future work should examine the motivations for conforming to well-liked peers using, for instance, experimental studies. This might shed light on possible mechanisms explaining contagion processes, expectations regarding rewarding outcomes and assess whether adopting the prosocial behavior of liked peers is motivated by a desire to improve one’s status or to form more social connections with peers who are kind and cooperative.

### Strengths, Limitations, and Future Directions

The strengths of this novel study are worth noting. First, the use of a Bayesian longitudinal social network analysis with three waves of data made it possible to test prosocial influence processes while controlling for selection effects. Failure to account for selection processes may lead to an overestimation of peer influence (Veenstra & Laninga-Wijnen, 2022), meaning that the behavior of the nominees could have also been explained by underlying homophily processes. Second, this study was the first to apply a social network design to test whether adolescents adopt the prosocial behaviors of the classmates they like. Thus, this study significantly adds to the existing literature by identifying a specific source of influence: the role of highly liked classmates within a network. Even though studies that examined prosocial influence are increasing, they have mainly used methodological approaches that do not allow the identification of students who are more influential or more susceptible to being influenced regarding prosociality.

This study also has some limitations. First, the number of nominations for liked peers was limited to three classmates. The main reason for this was to force participants to be more selective in their answers and prevent them from nominating all peers in their classroom. However, it is also a limitation since the number of students in each classroom was relatively large (ranging from 36 to 45), and therefore, some participants might have obtained more liking preferences if unlimited nominations had been allowed, affecting their likeability indegree score. In addition, for reasons associated with the cultural context, the sociometric question used in this study was an approximation of peers’ likeability. In the Chilean context, it is not possible to ask directly, “Who do you like most?” (the most common measure of peer likeability/acceptance) because it has a romantic connotation. Instead, students were asked about their possible preferences for hanging out, which was the best approximation for likeability/acceptance and has been used in earlier studies on peer relations in this context (Berger et al., 2015; Berger & Rodkin, 2012; Palacios et al., 2019). It does correlate moderately and positively with prosocial behavior and negatively with aggressive behavior, which is consistent with what other studies have found with the “who do you like most” operationalization. It is worth noting that prior studies with Chilean adolescents have yielded findings that are very similar to those obtained with American and European samples regarding behavioral influence (Berger & Rodkin, 2012; Dijkstra & Berger, 2018). However, the present study did not control for the potential overlap between likeability and popularity. Future studies could take into consideration this overlap to assess to what extent prosocial influence based on liking ties differs from those based on popularity ties.

Finally, the current study did not test any cognitive mechanism that might underlie prosocial influence processes. Although it is assumed that a desire for acceptance from liked peers was likely to be at play, other explanations are possible. In cases when participants interact with the peers they like, the influence for prosocial behaviors might simply reflect mutual reinforcement, which dictates that people who have been the recipient of favors or nice behaviors, behave in the same way towards the actor of such behaviors (Whatley et al., 1999). It is also possible that when youth witness a lot of prosocial behavior among the peers they like (and especially when others like these peers, too), they feel pressured to behave similarly to avoid rejection from peers and not necessarily to gain more status. One avenue for future research would be to disentangle underlying motivational processes associated with the spread of prosociality within a peer network.

### Practical Implications

The findings of this study have several important practical implications for educational interventions aiming to promote positive youth development, with a particular interest in fostering prosocial behaviors among students. Targeting high-status and highly connected students in educational interventions (e.g., social referent seeds) has been shown to be effective in diminishing maladaptive behaviors such as school conflict and encouraging others to take a public stance against different forms of school conflict (Paluck et al., 2016). The current findings highlight the potential of highly liked and prosocial adolescents to be involved in campaigns aiming to increase prosocial behavior. Interventions could indirectly contribute to improving prosocial attitudes and behaviors by effectively targeting a key source of influence who would work as a role model. Additionally, teachers and practitioners in schools might consider manipulating classroom seating arrangements to facilitate increases in peer acceptance among less-liked students. Seating arrangements (e.g., pairing high-status peers with low-status) have already been tested in a randomized control trial and found to improve peer acceptance and reduce externalizing behavior (van den Berg & Stoltz, 2018). The current study suggests that pairing less-liked youth with highly liked, prosocial peers within classrooms, might help their social integration and boost their own prosocial behavior in daily interactions.

## Conclusion

Peers that adolescents like have an important influence on their prosocial behavior, especially if these peers are also well-liked by other classmates. Although the influence was more often toward lower prosociality, adolescents who were relatively high on prosocial behavior, to begin with, also increased their prosociality when the peers they liked were even more prosocial. Importantly, the prosocial influence in an upward direction was especially driven by highly liked-prosocial peers, confirming a contagion process based on most-liked ties. In addition, less-liked peers were found particularly susceptible to this influence. Researchers and educators might consider the effect of well-liked, prosocial, and connected peers when developing educational programs or establishing policies to foster adolescents’ prosocial behavior. This is especially important to support those young adolescents who lack prosocial skills (or have difficulties regulating their emotions). These adolescents should be provided with opportunities to develop social skills. To this aim, well-liked peers can play a significant role as positive social referents to teach prosocial skills among peer classmates.

## Data Availability

The datasets generated and/or analyzed for the current study are not publicly available but are available from the corresponding author upon reasonable request.

## Appendix

**Appendix A**

Table 3

**Table 3:** Prior Mean Information Comparison

Parameters	Model 1 WITH Prior					Model 1 WITHOUT prior				
	Est.	SD	*p*	95% CI		Est.	SD(ƞ)	*p*	95% CI	
*Selection Effects*										
Outdegree (density) (ƞ)	−1.90	0.14	0.00	−2.19	−1.63	−1.97	0.59	0.00	−3.18	−0.81
Reciprocity (ƞ)	1.04	0.11	>0.99	0.80	1.29	1.05	0.60	0.96	−0.14	2.28
Transitivity (gwespf FF) (ƞ)	1.20	0.11	>0.99	0.99	1.41	1.15	0.59	0.98	0.04	2.30
Indegree-popularity (ƞ)	−0.02	0.06	0.35	−0.15	0.10	−0.06	0.55	0.47	−1.08	1.06
Outdegree-popularity (ƞ)	−0.07	0.08	0.19	−0.21	0.09	−0.03	0.58	0.48	−1.10	1.12
**Prosociality alter** (μ)	**0.22**	**0.03**	**>0.99**	**0.16**	**0.29**	**0.22**	**0.03**	**>0.99**	**0.15**	**0.28**
Prosociality ego (μ)	−0.06	0.03	0.01	−0.12	−0.01	−0.06	0.03	0.01	−0.11	−0.01
Prosociality similarity ego*alter (μ)	0.06	0.02	>0.99	0.02	0.11	0.05	0.02	>0.99	0.01	0.10
Sex alter (ƞ)	0.13	0.09	0.92	−0.05	0.32	0.14	0.55	0.60	−0.96	1.25
Sex ego (ƞ)	0.01	0.10	0.54	−0.18	0.21	0.04	0.57	0.54	−1.16	1.17
Same sex (ƞ)	0.32	0.11	>0.99	0.11	0.53	0.33	0.59	0.72	−0.76	1.52
*Influence effects: Prosocial behavior*										
Prosociality linear shape (ƞ)	−0.51	0.23	0.01	−0.94	−0.06	−1.27	0.82	0.06	−2.95	0.37
Prosociality quadratic shape (ƞ)	−0.27	0.09	0.00	−0.46	−0.09	−0.57	0.58	0.15	−1.68	0.59
Prosociality indegree (ƞ)	0.22	0.11	0.98	0.01	0.42	0.46	0.61	0.77	−0.67	1.71
Sex (ƞ)	−0.37	0.19	0.03	−0.74	0.01	−0.74	0.72	0.16	−2.18	0.55
**Prosociality average alter** (μ)	**0.64**	**0.20**	**>0.99**	**0.25**	**1.05**	**1.82**	**0.57**	**>0.99**	**0.77**	**3.03**

Reasonable values were chosen for the prior means based on previous research on liking adolescents’ networks (Mikami & Mercer, 2017; Sentse et al., 2014; Van Ryzin et al., 2016). For the outdegree parameter, a value of –2 was chosen, reflecting the scarcity of liking networks; for the reciprocity, transitivity (GWESPFF), and same-sex parameters, a value of +1 was chosen, indicating that those effects are likely to occur. The results of those priors and an analogous model were compared without prior mean information for the first model. The rows in bold represent the estimates regarding the main selection (prosociality alter) and influence effect (prosociality average alter) associated with prosocial behavior.

**Appendix B**

Table 4

**Table 4:** Model Specification of Parameters

Parameter	Description
*Network Structures*	
Outdegree (density)	General tendency to select others as *liked* peers
Reciprocity (recip)	Tendency toward reciprocation
Transitivity ((GWESP I −>K −>J (69)))	Tendency to form triads or transitive group formation
Outdegree activity (outact)	Tendency representing those youth who give many nominations will give more nominations over time.
indegree – popularity (inpop)	Reflect youth who receive many nominations tend to receive more nominations over time
outdegree – popularity (outpop)	Reflects youth who give many nominations to give more nominations over time
*Selection Effects*	
Ego effect for prosociality (egoX)	Prosocial youth have a higher tendency to nominate more liked peers
Alter effect for prosociality (altX)	Prosocial youth have a higher tendency to be nominated as liked by peers
Similarity Ego*Alter (egoXaltX)	Youth similar in prosocial behavior are likely to select each other as liked peers.
Ego effect for sex (egoX)	Tendency to nominate like peers based on their sex
Alter effect for sex (alterX)	Tendency to be nominated as liked by peers based on their sex
Similarity in sex (sameX)	Tendency to *like* same-sex peers
*Influence Effects*	
Prosocial linear shape (linear)	The two parameters together define a parabola shape of the objective function, allowing it to capture the basic shape of the observed distribution of the behavioral variable (Steglich et al., 2010). Linear shape reflects the tendency of prosociality to linearly increase over time. Quadratic shape reflects the tendency of prosociality to increase then decrease over time
Prosocial quadratic shape (quad)	
Average alter effect (avAlt)	*Main effect of liked adolescents on peers’ prosocial behavior*: Tendency of youth to change their prosocial behavior in response to *liked* peers’ prosocial behavior
Prosocial: effect from sex	Tendency toward high prosocial behavior based on the sex of peers
Prosocial: indegree effect	Actors receiving more liking nominations (a higher indegree) have a stronger tendency toward high prosocial behavior
Prosocial: outdegree effect	More active actors (with a higher outdegree) have a stronger tendency toward high prosocial behavior
Average Alter× *popAlt* (avAltPop)	Interaction effect for social reinforcement: tendency of youth to change their prosocial behavior when their liked peers are also liked by many others
Average Alter x *indegree likeability*	Interaction effect for susceptibility to peer influence on prosocial behavior: tendency of youth to change their prosocial behavior based on their own liking scores

**Appendix C**

Table 5

**Table 5:** Ego-Alter Influence Table: Influence of Liked Peers (Alters) on Youth’s (Ego’s) Prosocial Behavior

Average prosocial behavior of liked peers (Alters)	Adolescents’ Prosocial Behavior (Ego)				
	0	1	2	3	4
0	1.94	1.00	−0.49	−2.51	−5.07
1	0.86	0.55	−0.30	−1.69	−3.61
2	−0.23	0.10	−0.12	−0.87	−2.16
3	−1.32	−0.36	0.07	−0.05	−0.70
4	−2.40	−0.81	0.25	0.77	0.75

Values in the table represent log odds. Sample sizes are *n* = 294 at Wave 1, *n* = 282 at Wave 2, and *n* = 275 at Wave 3. Numbers in the table reflect the strength of peer influence for youth to change their prosocial behavior based on liked peers’ average levels of prosociality (columns dependent on rows). The values in the diagonal indicate the likelihood of liked peers' influence to occur when adolescents have exactly the same score on prosociality. Comparing the values between rows and columns, it is possible to see that the influence of prosociality based on liked peers occurs among adolescents. This influence mostly developed towards lower values of prosocial behavior (negative trend). However, youth who are, to some extent prosocial (i.e., 2, 3, and 4) were more likely to develop higher prosociality themselves when they nominated liked peers with the highest prosocial behavior scores (i.e., 3 or 4) than were youth with the lowest prosocial behavior scores (i.e., 0 or 1). Lowly prosocial youth (i.e., 0) developed higher prosociality themselves when they nominated liked peers with similar scores on prosociality (i.e., 0) and also 1. While those adolescents scoring 1 developed higher values of prosociality themselves by nominating liked peers with a 1 and 2 average prosocial behavior

## Footnotes

1 In the Chilean context, it is not possible to ask directly, “Who do you like most?” as many other studies have done for measuring peer likeability/acceptance. The main reason is that in this context, it has a romantic connotation, and instead, students were asked about their preferences for hanging out, which was the best approximation for likeability/acceptance in this context.

## References

[CR1] Adler, P. A., & Adler, P. (1995). Dynamics of inclusion and exclusion in preadolescent cliques. *Social Psychology Quarterly*, *58*(3), 145–162. 10.2307/2787039.

[CR2] Ahmed, S., Foulkes, L., Leung, J. T., Griffin, C., Sakhardande, A., Bennett, M., Dunning, D. L., Griffiths, K., Parker, J., Kuyken, W., Williams, M. G., Dalgleish, T., & Blakemore, S. J. (2020). Susceptibility to prosocial and antisocial influence in adolescence. *Journal of Adolescence*, *84*(1), 56–68. 10.1016/j.adolescence.2020.07.012. 32858504 10.1016/j.adolescence.2020.07.012 PMC7674583

[CR3] Bandura, A., & Walters, R. H. (1977). *Social Learning Theory (Vol. 1).* Prentice-Hall.

[CR4] Baumeister, R. F., & Leary, M. R. (1995). The need to belong: Desire for interpersonal attachments as a fundamental human motivation. *Psychological Bulletin*, *117*(3), 497–529. 10.1037/0033-2909.117.3.497. 7777651

[CR5] Berger, C., & Rodkin, P. (2012). Group influences on individual aggression and prosociality: early adolescents who change peer affiliations. *Social Development*, *21*(2), 396–413. 10.1111/j.1467-9507.2011.00628.x.

[CR6] Berger, C., Batanova, M., & Cance, J. D. (2015). Aggressive and prosocial? Examining latent profiles of behavior, social status, machiavellianism, and empathy. *Journal of Youth and Adolescence*, *44*(12), 2230–2244. 10.1007/s10964-015-0298-9. 25987411 10.1007/s10964-015-0298-9

[CR7] Bowker, A. (2004). Predicting friendship stability during early adolescence. *Journal of Early Adolescence*, *24*, 85–112. 10.1177/0272431603262666.

[CR8] Brechwald, W. A., & Prinstein, M. J. (2011). Beyond homophily: a decade of advances in understanding peer influence processes. *Journal of Research on Adolescence*, *21*(1), 166–179. 10.1111/j.1532-7795.2010.00721.x. 23730122 10.1111/j.1532-7795.2010.00721.x PMC3666937

[CR9] Caprara, G. V., Barbaranelli, C., Pastorelli, C., Bandura, A., & Zimbardo, P. G. (2000). Prosocial foundations of children’s academic achievement. *Psychological Science*, *11*(4), 302–306. 10.1111/1467-9280.00260. 11273389 10.1111/1467-9280.00260

[CR10] Carlo, G., Crockett, L. J., Randall, B. A., & Roesch, S. C. (2007). A latent growth curve analysis of prosocial behavior among rural adolescents. *Journal of Research on Adolescence*, *17*, 301–324. 10.1111/j.1532-7795.2007.00524.x.

[CR11] Ch vez, D. V., Salmivalli, C., Garandeau, C. F., Berger, C. & Luengo Kanacri, B. P. (2022). Bidirectional associations of prosocial behavior with peer acceptance and rejection in adolescence. *Journal of Youth and Adolescence*, *51*(12), 2355–2367. 10.1007/s10964-022-01675-5. 36114945 10.1007/s10964-022-01675-5 PMC9596566

[CR12] Chartrand, T. L., & Bargh, J. A. (1999). The chameleon effect: the perception–behavior link and social interaction. *Journal of Personality and Social Psychology*, *76*(6), 893–910. 10.1037/0022-3514.76.6.893. 10402679 10.1037//0022-3514.76.6.893

[CR13] Cho, H. J., & Hackel, L. M. (2022). Instrumental learning of social affiliation through outcome and intention. *Journal of Experimental Psychology: General*, *151*(9), 2204–2221. 10.1037/xge0001190. 35446107 10.1037/xge0001190

[CR14] Choukas-Bradley, S., Giletta, M., Cohen, G. L., & Prinstein, M. J. (2015). Peer influence, peer status, and prosocial behavior: an experimental investigation of peer socialization of adolescents’ intentions to volunteer. *Journal of Youth and Adolescence*, *44*(12), 2197–2210. 10.1007/s10964-015-0373-2. 26525387 10.1007/s10964-015-0373-2 PMC5985442

[CR15] Cillessen, A. H. N., & Rose, A. J. (2005). Understanding popularity in the peer system. *Current Directions in Psychological Science*, *14*(2), 102–105. 10.1111/j.0963-7214.2005.00343.x.

[CR16] Costello, B. J., & Zozula, C. (2018). Peer influence: mechanisms and motivations. *Deviant Behavior*, *39*(1), 94–110. 10.1080/01639625.2016.1260387.

[CR17] Crone, E. A., & Achterberg, M. (2022). Prosocial development in adolescence. *Current Opinion in Psychology*, *44*, 220–225. 10.1016/j.copsyc.2021.09.020. 34749238 10.1016/j.copsyc.2021.09.020

[CR18] Crone, E. A., Sweijen, S. W., Te Brinke, L. W., & van de Groep, S. (2022). Pathways for engaging in prosocial behavior in adolescence. *Advances in Child Development and Behavior*, *63*, 149–190. 10.1016/bs.acdb.2022.03.003. 35871821 10.1016/bs.acdb.2022.03.003

[CR19] de Bruine, M., Giletta, M., Denissen, J. J. A., Sijtsema, J. J., & Oldehinkel, A. J. (2019). A healthy peer status: peer preference, not popularity, predicts lower systemic inflammation in adolescence. *Psychoneuroendocrinology*, *109*, 104402 10.1016/j.psyneuen.2019.104402. 31465942 10.1016/j.psyneuen.2019.104402

[CR20] DeLay, D., Burk, W. J., & Laursen, B. (2022). Assessing peer influence and susceptibility to peer influence using individual and dyadic moderators in a social network context: the case of adolescent alcohol misuse. *International Journal of Behavioral Development*, *46*(3), 208–221. 10.1177/01650254221084102. 35645435 10.1177/01650254221084102 PMC9139630

[CR21] Dijkstra, J. K., & Berger, C. (2018). Friendship Selection And Influence Processes For Physical Aggression And Prosociality: Differences Between Single-sex And Mixed-sex Contexts. *Sex Roles*, *78*(9–10), 625–636. 10.1007/S11199-017-0818-Z/TABLES/3. 29670316 10.1007/s11199-017-0818-z PMC5897469

[CR22] Dijkstra, J. K., Cillessen, A. H. N., Lindenberg, S., & Veenstra, R. (2010). Basking in reflected glory and its limits: why adolescents hang out with popular peers. *Journal of Research on Adolescence*, *20*(4), 942–958. 10.1111/j.1532-7795.2010.00671.x.

[CR23] Eisenberg, N., Spinrad, T. L., & Knafo-Noam, A. (2015). Prosocial development. In M. E. Lamb, & R. M. Lerner (Eds.), *Handbook of child psychology and developmental science: socioemotional processes* (Vol. 3, 7th ed., pp. 610–656). Wiley.

[CR24] Faur, S., Laursen, B., & Juvonen, J. (2022). Adolescents with few friend alternatives are particularly susceptible to influence from friends. *Journal of Youth and Adolescence*, *52*, 637–650. 10.1007/s10964-022-01718-x. 36484895 10.1007/s10964-022-01718-x

[CR25] Field, N. H., Choukas‐Bradley, S., Giletta, M., Telzer, E. H., Cohen, G. L., & Prinstein, M. J. (2023). Why adolescents conform to high‐status peers: associations among conformity, identity alignment, and self‐esteem. *Child Development,*10.1111/cdev.14038. 10.1111/cdev.14038 PMC11023764 37966044

[CR26] Findley-Van Nostrand, D., & Ojanen, T. (2018). Forms of prosocial behaviors are differentially linked to social goals and peer status in adolescents. *The Journal of Genetic Psychology*, *179*(6), 329–342. 10.1080/00221325.2018.1518894. 30346917 10.1080/00221325.2018.1518894

[CR27] Foulkes, L., Leung, J. T., Fuhrmann, D., Knoll, L. J., & Blakemore, S.-J. (2018). Age differences in the prosocial influence effect. *Developmental Science*, *21*(6), 1–9. 10.1111/desc.12666. 10.1111/desc.12666 PMC6221149 29658168

[CR28] Gelman, A., Hwang, J., & Vehtari, A. (2014). Understanding predictive information criteria for Bayesian models. *Statistics and Computing*, *24*, 997–1016. 10.1007/s11222-013-9416-2.

[CR29] Gommans, R., Sandstrom, M. J., Stevens, G. W. J. M., ter Bogt, T. F. M., & Cillessen, A. H. N. (2017). Popularity, likeability, and peer conformity: four field experiments. *Journal of Experimental Social Psychology*, *73*, 279–289. 10.1016/j.jesp.2017.10.001.

[CR30] Hartup, W. W., & Stevens, N. (1997). Friendships and adaptation in the life course. *Psychological Bulletin*, *121*(3), 355–370. 10.1037/0033-2909.121.3.355.

[CR31] Huisman, M. E., & Steglich, C. (2008). Treatment of non-response in longitudinal network data. *Social Networks*, *30*(4), 297–308. 10.1016/j.socnet.2008.04.004.

[CR32] Kwon, K., & Lease, A. M. (2014). Perceived influence of close friends, well-liked peers, and popular peers. *Journal of Social and Personal Relationships*, *31*(8), 1116–1133. 10.1177/0265407514522887.

[CR33] Laninga‐Wijnen, L., Harakeh, Z., Steglich, C., Dijkstra, J. K., Veenstra, R., & Vollebergh, W. (2017). The norms of popular peers moderate friendship dynamics of adolescent aggression. *Child Development*, *88*(4), 1265–1283. 10.1111/cdev.12650. 27779756 10.1111/cdev.12650

[CR34] Laninga‐Wijnen, L., Harakeh, Z., Garandeau, C. F., Dijkstra, J. K., Veenstra, R., & Vollebergh, W. A. M. (2019). Classroom popularity hierarchy predicts prosocial and aggressive popularity norms across the school year. *Child Development*, *90*(5), 637–653. 10.1111/cdev.13228. 10.1111/cdev.13228 PMC6849822 30825397

[CR35] Laninga-Wijnen, L., & Veenstra, R. (2021). Peer similarity in adolescent social networks: types of selection and influence, and factors contributing to openness to peer influence. In B. Halpern-Felsher (Ed.) *The encyclopedia of child and adolescent health*. Elsevier.

[CR36] Laursen, B., & Faur, S. (2022). What does it mean to be susceptible to influence? A brief primer on peer conformity and developmental changes that affect it. *International Journal of Behavioral Development*, *46*(3), 222–237. 10.1177/01650254221084103. 35990791 10.1177/01650254221084103 PMC9387868

[CR37] Laursen, B., Leggett-James, M. P., & Valdes, O. M. (2023). Relative likeability and relative popularity as sources of influence in children’s friendships. *PLoS ONE*, *18*(5), e0283117 10.1371/journal.pone.0283117. 37172045 10.1371/journal.pone.0283117 PMC10180626

[CR38] Laursen, B., & Veenstra, R. (2023). In defense of peer influence: the unheralded benefits of conformity. *Child Development Perspectives*, *17*(1), 74–80. 10.1111/cdep.12477.

[CR39] Layous, K., Nelson, S. K., Oberle, E., Schonert-Reichl, K. A., & Lyubomirsky, S. (2012). Kindness Counts: prompting prosocial behavior in preadolescents boosts peer acceptance and well-being. *PLoS ONE*, *7*(12), e51380. 10.1371/journal.pone.0051380. 23300546 10.1371/journal.pone.0051380 PMC3530573

[CR40] Logis, H., Rodkin, P. C., Gest, S. D., & Ahn, H.-J. (2013). Popularity as an organizing factor of preadolescent friendship networks: beyond prosocial and aggressive behavior. *Journal of Research on Adolescence*, *16*, 119–128. 10.1111/jora.12033.

[CR41] Luengo Kanacri, B. P., & Jiménez-Moya, G. (2017). Good practices on civic engagement in Chile and the role of promoting prosocial behaviors in school settings. In B. García-Cabrero, A. Sandoval-Hernández, E. Treviño-Villareal, S. D. Ferráns, M. G. P. Martínez (Eds.), *Civics and citizenship* (pp. 241–254). Sense Publishers.

[CR42] Luengo Kanacri, B. P., Pastorelli, C., Eisenberg, N., Zuffianò, A., & Caprara, G. V. (2013). The development of prosociality from adolescence to early adulthood: the role of effortful control. *Journal of Personality*, *81*(3), 302–312. 10.1111/jopy.12001. 22924862 10.1111/jopy.12001

[CR43] Luengo Kanacri, B. P., Zuffiano, A., Pastorelli, C., Jiménez‐Moya, G., Tirado, L. U., Thartori, E., Gerbino, M., Cumsille, P., & Martinez, M. L. (2020). Cross‐national evidences of a school‐based universal programme for promoting prosocial behaviours in peer interactions: main theoretical communalities and local unicity. *International Journal of Psychology*, *55*(S1), 48–59. 10.1002/ijop.12579. 31232475 10.1002/ijop.12579

[CR44] Markievicz, D., Brendgen, M., Doyle, A. B., & Bukowski, W. M. (2001). The relations between friendship quality, ranked-friendship preference, and adolescents’ behavior with their friends. *Merrill-Palmer Quarterly*, *47*(3), 395–415. http://www.jstor.org/stable/23093405

[CR45] Memmott-Elison, M. K., Holmgren, H. G., Padilla-Walker, L. M., & Hawkins, A. J. (2020). Associations between prosocial behavior, externalizing behaviors, and internalizing symptoms during adolescence: a meta-analysis. *Journal of Adolescence*, *80*(1), 98–114. 10.1016/j.adolescence.2020.01.012. 32087386 10.1016/j.adolescence.2020.01.012

[CR46] Mikami, A. Y., & Mercer, S. H. (2017). Teacher behaviors toward children with attention-deficit/hyperactivity disorder predict peers’ initial liking and disliking impressions in a summer camp setting. *Journal of Social and Clinical Psychology*, *36*(6), 506–534. 10.1521/jscp.2017.36.6.506.

[CR47] Molano, A., Jones, S. M., Brown, J. L., & Aber, J. L. (2013). Selection and socialization of aggressive and prosocial behavior: the moderating role of social‐cognitive processes. *Journal of Research on Adolescence*, *23*(3), 424–436. 10.1111/jora.12034.

[CR48] Palacios, D., Berger, C., Luengo Kanacri, B. P., Veenstra, R., & Dijkstra, J. K. (2019). The interplay of adolescents’ aggression and victimization with friendship and antipathy networks within an educational prosocial intervention. *Journal of Youth and Adolescence*, *48*(10), 2005–2022. 10.1007/s10964-019-01105-z. 31482513 10.1007/s10964-019-01105-z PMC6813759

[CR49] Paluck, E. L., Shepherd, H., & Aronow, P. M. (2016). Changing climates of conflict: a social network experiment in 56 schools. *Proceedings of the National Academy of Sciences USA*, *113*(3), 566–571. 10.1073/pnas.1514483113. 10.1073/pnas.1514483113 PMC4725542 26729884

[CR50] Park, S., & Shin, J. (2017). The influence of anonymous peers on prosocial behavior. *PLoS ONE*, *12*(10), e0185521 10.1371/journal.pone.0185521. 29016612 10.1371/journal.pone.0185521 PMC5633145

[CR51] Parkhurst, J. T., & Hopmeyer, A. (1998). Sociometric popularity and peer-perceived popularity: two distinct dimensions of peer status. *The Journal of Early Adolescence*, *18*(2), 125–144. 10.1177/0272431698018002001.

[CR52] Powers, K. E., Somerville, L. H., Kelley, W. M., & Heatherton, T. F. (2013). Rejection sensitivity polarizes striatal-medial prefrontal activity when anticipating social feedback. *Journal of Cognitive Neuroscience*, *25*(11), 1887–1895. 10.1162/jocn_a_00446. 23859650 10.1162/jocn_a_00446 PMC4076412

[CR53] Rambaran, A. J., Dijkstra, J. K., & Stark, T. H. (2013). Status-based influence processes: the role of norm salience in contagion of adolescent risk attitudes. *Journal of Research on Adolescence*, *23*(3), 574–585. 10.1111/jora.12032.

[CR54] Ripley, R. M., Snijders, T. A. B., Bóda, Z., Vörös, A., & Preciado, P. (2023). *Manual for Siena version 4.0.* University of Oxford Department of Statistics, Nuffield College.

[CR55] Sentse, M., Kiuru, N., Veenstra, R., & Salmivalli, C. (2014). A social network approach to the interplay between adolescents’ bullying and likeability over time. *Journal of Youth and Adolescence*, *43*(9), 1409–1420. 10.1007/s10964-014-0129-4. 24752280 10.1007/s10964-014-0129-4

[CR56] Shin, H. (2017). Friendship dynamics of adolescent aggression, prosocial behavior, and social status: the moderating role of gender. *Journal of Youth and Adolescence*, *46*(11), 2305–2320. 10.1007/s10964-017-0702-8. 28699121 10.1007/s10964-017-0702-8

[CR57] Snijders, T. A. B., & Lomi, A. (2019). Beyond homophily: incorporating actor variables in statistical network models. *Network Science*, *7*(1), 1–19. 10.1017/nws.2018.30.

[CR58] Snijders, T. A. B., van de Bunt, G. G., & Steglich, C. E. G. (2010). Introduction to stochastic actor-based models for network dynamics. *Social Networks*, *32*(1), 44–60. 10.1016/j.socnet.2009.02.004.

[CR59] Steglich, C., Snijders, T. A. B., & Pearson, M. (2010). Dynamic networks and behavior: separating selection from influence. *Sociological Methodology*, *40*(1), 329–393. 10.1111/j.1467-9531.2010.01225.x.

[CR60] Stotsky, M. T., & Bowker, J. C. (2018). An examination of reciprocal associations between social preference, popularity, and friendship during early adolescence. *Journal of Youth and Adolescence*, *47*, 1830–1841. 10.1007/s10964-018-0846-1. 29616384 10.1007/s10964-018-0846-1

[CR61] Telzer, E. H., van Hoorn, J., Rogers, C. R., & Do, K. T. (2018). Social influence on positive youth development: a developmental neuroscience perspective. In *Advances in child development and behavior* (pp. 215–258). 10.1016/bs.acdb.2017.10.003. 10.1016/bs.acdb.2017.10.003 PMC6345387 29455864

[CR62] Thomas, K. K., & Bowker, J. C. (2013). An investigation of desired friendships during early adolescence. *The Journal of Early Adolescence*, *33*(6), 867–890. 10.1177/0272431612469725.

[CR63] van den Berg, Y. H. M., Lansu, T. A. M., & Cillessen, A. H. N. (2020). Preference and popularity as distinct forms of status: a meta‐analytic review of 20 years of research. *Journal of Adolescence*, *84*(1), 78–95. 10.1016/j.adolescence.2020.07.010. 32891019 10.1016/j.adolescence.2020.07.010

[CR64] van den Berg, Y. H. M., & Stoltz, S. (2018). Enhancing Social Inclusion of Children With Externalizing Problems Through Classroom Seating Arrangements: A Randomized Controlled Trial. *Journal of Emotional and Behavioral Disorders*, *26*(1), 31–41. 10.1177/1063426617740561. 29503518 10.1177/1063426617740561 PMC5815425

[CR65] Van Ryzin, M., DeLay, D., & Dishion, T. J. (2016). Being well-liked predicts increased use of alcohol but not tobacco in early adolescence. *Addictive Behaviors*, *53*, 168–174. 10.1016/j.addbeh.2015.10.017. 26547042 10.1016/j.addbeh.2015.10.017 PMC4679564

[CR66] Veenstra, R., Dijkstra, J. K., Steglich, C., & Van Zalk, M. H. W. (2013). Network-behavior dynamics. *Journal of Research on Adolescence*, *23*, 399–412. 10.1111/jora.12070.

[CR67] Veenstra, R., & Laninga-Wijnen, L. (2022). Peer network studies and interventions in adolescence. *Current Opinion in Psychology*, *44*, 157–163. 10.1016/j.copsyc.2021.09.015. 34662775 10.1016/j.copsyc.2021.09.015

[CR68] Whatley, M. A., Webster, J. M., Smith, R. H., & Rhodes, A. (1999). The effect of a favor on public and private compliance: how internalized is the norm of reciprocity? *Basic and Applied Social Psychology*, *21*(3), 251–259. 10.1207/S15324834BASP2103_8.

